# Histone lactylation in macrophages is predictive for gene expression changes during ischemia induced-muscle regeneration

**DOI:** 10.1016/j.molmet.2024.101923

**Published:** 2024-03-22

**Authors:** Thibaut Desgeorges, Eva Galle, Jing Zhang, Ferdinand von Meyenn, Katrien De Bock

**Affiliations:** 1 Laboratory of Exercise and Health, Institute of Human Movement Sciences and Sport, Department of Health Sciences and Technology, ETH Zurich, Zurich, Switzerland; 2 Laboratory of Nutrition and Metabolic Epigenetics, Institute for Food, Nutrition and Health, Department of Health Sciences and Technology, ETH Zurich, Zurich, Switzerland

**Keywords:** Inflammation, Macrophage, Muscle regeneration, Lactylation

## Abstract

**Objectives:**

We have previously shown that lactate is an essential metabolite for macrophage polarisation during ischemia-induced muscle regeneration. Recent *in vitro* work has implicated histone lactylation, a direct derivative of lactate, in macrophage polarisation. Here, we explore the *in vivo* relevance of histone lactylation for macrophage polarisation after muscle injury.

**Methods:**

To evaluate macrophage dynamics during muscle regeneration, we subjected mice to ischemia-induced muscle damage by ligating the femoral artery. Muscle samples were harvested at 1, 2, 4, and 7 days post injury (dpi). CD45^+^CD11b^+^F4/80^+^CD64^+^ macrophages were isolated and processed for RNA sequencing, Western Blotting, and CUT&Tag-sequencing to investigate gene expression, histone lactylation levels, and histone lactylation genomic localisation and enrichment, respectively.

**Results:**

We show that, over time, macrophages in the injured muscle undergo extensive gene expression changes, which are similar in nature and in timing to those seen after other types of muscle-injuries. We find that the macrophage histone lactylome is modified between 2 and 4 dpi, which is a crucial window for macrophage polarisation. Absolute histone lactylation levels increase, and, although subtly, the genomic enrichment of H3K18la changes. Overall, we find that histone lactylation is important at both promoter and enhancer elements. Lastly, H3K18la genomic profile changes from 2 to 4 dpi were predictive for gene expression changes later in time, rather than being a reflection of prior gene expression changes.

**Conclusions:**

Our results suggest that histone lactylation dynamics are functionally important for the function of macrophages during muscle regeneration.

## Highlights

• Macrophages recruited to the injured ischemic muscle display extensive gene changes over time.
• The macrophage lactylome is modified during macrophage polarization.
• Lactylation in macrophages occurs at both promoter and enhancer elements and is predictive of gene expression overtime.

## 1 Introduction

Skeletal muscle regeneration is dependent on the interplay of various cell types, including resident muscle stem cells, the vasculature and several immune cell types like macrophages [[1], [2], [3], [4]]. Upon muscle damage such as during ischemia, muscle disease, or toxin induced injury, muscle stem cells exit their quiescent state, get activated and give rise to a population of proliferating myogenic progenitor cells that will ultimately differentiate and fuse with each other or with remaining myofibers to repair the damaged muscle [2,5]. Immune cells play a critical role in this process both as damage sensors as well as effector cells and a dysregulated immune response impairs skeletal muscle regeneration [6]. In particular, monocyte-derived lymphocyte antigen 6 expressing (Ly6C^hi^) macrophages in the injured muscle initially exhibit an inflammatory phenotype through the expression of specific pro-inflammatory genes, such as *Tlr4*, *Ifn1b* and interleukins *Il15* and *Il6,* as well as through the expression and secretion of cytokines such as TNF-α and IL-1β [1,[7], [8], [9]]. Inflammatory macrophages also promote muscle stem cell proliferation, induce the apoptosis of activated fibroadipogenic progenitors [10], and contribute to removing necrotic debris through efferocytosis [11]. Soon thereafter, they lose Ly6C expression and functionally repolarize towards a restorative phenotype that continues to remove necrotic debris. At the same time, Ly6C^lo^ restorative macrophages actively support muscle regeneration through the secretion of growth factors such as IGF1, GDF3, GDF15, and TGFβ that promote the differentiation and fusion of committed muscle progenitor cells [1,[7], [8], [9],^12^,^13^]. They do so through steering the differentiation of fibroadipogenic progenitors towards fibroblasts that deposit an extracellular matrix [6,14], and by stimulating the growth of new blood vessels through secreting the angiogenic factor VEGF [3,15,16]. This sequence of events is tightly controlled, strictly timed and imperative for proper tissue recovery [17], but the mechanisms that control macrophage function and functional repolarization are incompletely understood and likely dependent on cell intrinsic changes, cell–cell interactions, as well as the tissue microenvironment in which they reside.

Besides different functional properties, inflammatory and restorative macrophages have different metabolic properties and those are critical for their effector functions [18]. Inflammatory macrophages are highly glycolytic but, during their repolarization towards a restorative phenotype, switch to a more oxidative phenotype [19]. This switch is dependent on the cellular energy sensor AMPK, and deleting AMPK prevents their repolarization [20]. High glycolysis is associated with the expression and secretion of inflammatory cytokines [19,21]. Efferocytosis initiation also activates glycolysis. At the same time, conditioned medium of efferocytic macrophages, high in lactate, contributes to an anti-inflammatory environment by promoting the activation of anti-inflammatory genes in naive macrophages [22]. In ischemia-induced damaged muscle, lactate derived from angiogenic endothelial cells promotes the metabolic and functional switch from an inflammatory towards a restorative phenotype [15], in a mechanism that is dependent on the uptake of lactate by macrophages. Little is however known about the intracellular pathways that are involved in coordinating the phenotype switch induced by lactate. One recent mechanism that has been proposed to exercise this signalling role of lactate is the modulation of the epigenome. Histone lactylation is a post-translational modification (PTM) of histone proteins, the core elements of nucleosomes. It involves the addition of lactate molecules to lysine residues in the histone tails which has been shown to modulate gene expression and the immune response in various contexts [23]. Lactate-derived lactylation of histones has been shown *in vitro* to induce M2-like genes in M1 Bone Marrow Derived Macrophages (BMDM) [23]. In the context of cancer, lactylation levels in tumour-associated macrophages correlated with the expression of *Arg1,* a gene essential for restorative macrophage function, but not *Vegfa* [23]. Mechanistically, lactate (either exogenous or glycolysis derived) increased Histone 3 lysine 18 lactylation (H3K18la) levels at the *Arg1* promoter as well as its gene expression, thereby increasing the expression of a set of restorative genes in inflammatory macrophages. Whether histone lactylation is dynamically regulated during macrophage fate transitions and functionally affects gene expression is not known. In this study, we decided to explore potential *in vivo* contributions of histone lactylation to macrophage gene regulation in ischemia-induced muscle damage.

## 2 Materials and methods

### 2.1 Animals

C57BL6J wild type males (Charles River) from 8 to 12 weeks old were used in this study. Animals were housed in a normal light cycle with *ad libitum* access to food and water. Hindlimb ischemia experiments were performed as described [15]. Briefly, mice were anesthetized with isoflurane. The hindlimb was shaved, and the skin was incised. The proximal end of the femoral artery and the distal portion of the saphenous artery were ligated. The artery and all side-branches were dissected to be freed and the femoral artery and attached side-branches were excised. Procedures were approved by the ethics committee (number ZH050-2021). To label circulating b(lood)-CD45^+^ cells, mice were anaesthetised with a combination of ketamine and xylazine and i.v. Injected with 3 μg of PE anti-mouse CD45 antibody (Biolegend [30-F11], 103106) diluted in PBS (as described in Mysore et al. [24] with minor modifications). Five minutes later, mice were sacrificed and muscle was processed for further analysis (see below). Immediately after sacrifice, blood samples were collected via intracardiac puncture. Blood was directly diluted in PBS containing heparin and was used as positive control for CD45 staining.

### 2.2 Western blot

Histone extracts were prepared with the acid histone extraction protocol published by Abcam. Histone protein extracts were resolved using a gradient SDS-PAGE before being immunoblotted onto a PVDF membrane. The membrane was blocked for 1 h in blocking solution (TBS/0.1% Tween/5% milk) and then incubated overnight at 4 °C with primary antibodies diluted in blocking solution. After washes with TBS/0.1% Tween, membranes were incubated with secondary antibodies conjugated with fluorescent or HRP tag diluted in blocking buffer for 1 h at room temperature. Band signals were visualised using Bio-Rad ChemiDoc Imaging System. The following primary antibodies were used: H3 (Abcam, ab1791), pan-KLA (PTM Bio, PTM-1401), and H3K18la (PTM Bio, PTM-1406 or PTM-1406RM). The secondary antibodies used were an HRP-conjugated monoclonal rabbit anti-rabbit IgG (Cell Signaling Technology, 7074s).

### 2.3 Macrophage isolation and immune cell staining

To isolate macrophages, calf muscle from the ischemic limb was collected and digested in 2 mg/mL Collagenase IV (Thermo Fisher Scientific, 17104019)/Dispase II (Sigma–Aldrich, D4693-1G) for 45 min to 1 h at 37 °C. After filtration and washing steps, red blood cells were removed with ACK Lysis buffer (Gibco, A1049201). For either histone isolation or CUT&Tag, t(issue)-CD45^+^CD11b^+^F4/80^+^CD64^+^ living macrophages (DAPI selection to remove debris, BD biosciences, 564907) were stained and sorted (Sony Cell sorter SH800S). The used antibodies were the following: PE anti-mouse CD45 (Biolegend [30-F11], 103106), PerCP/Cy5.5 anti-mouse/human CD11b (Biolegend [M1/70], 101228), Alexa Fluor® 488 anti-F4/80 mouse Monoclonal Antibody (Biolegend [clone: 30-F11], 103122), APC anti-CD64 mouse Monoclonal Antibody (Biolegend [clone: X54-5/7.1], 139306). For immune cell analysis, calf muscle from ischemic limb was collected and digested as described above and was stained for cytometry. Blood samples were also collected at the time of sacrifice and stained with the same protocol after one step of red blood cell lysis step (ACK buffer, Gibco, A1049201). The used antibodies were the following: viability dye efluor780 (Thermofisher, 65-0865-14), AF488 anti-mouse CD45 antibody (Biolegend, [clone: S18009D], 160306), PerCP/Cy5.5 anti-mouse/human CD11b (Biolegend [M1/70], 101228), APC anti-CD64 Mouse Monoclonal Antibody (Biolegend [clone: X54-5/7.1], 139306), BV711 anti-F4/80 mouse Monoclonal Antibody (Biolegend, [clone: BM8], 123147), BV650 anti-CD11c mouse Monoclonal Antibody (Biolegend, [clone: N418], 117339, BV421 anti-SiglecF mouse Monoclonal Antibody (Biolegend, [clone: S17007L], 155509), AF700 anti-mouse Ly6G antibody (Biolegend, [clone: 1A8], 127622), PE-cy7 anti-mouse Ly6C antibody (Biolegend, [clone: HK1.4], 128018). FACS analyses were done with the BD Fortesa device and analyzed with FlowJo software.

### 2.4 Muscle lactate measurement

Muscle tissue lactate concentration was determined using the Lactate-Glo Assay (Promega, J5021) according to the manufacturer's protocol.

### 2.5 RNA library preparation and sequencing

Total RNA for each sample was extracted using RNeasy micro kit (QIAGEN, 74106). Quantification of total RNA was performed using the Tecan microplate reader (DKSH). Quality control of RNA was performed with the Tapestation system (Agilent). 50–100 ng of RNA was used as an input for the Smart-seq2/3 protocol [[25], [26], [27]] including cDNA synthesis (quantified with the Qubit system for further steps), pre-amplification, tagmentation, and enrichment step. In short, RNA libraries were prepared by performing reverse transcription and template switching using Maxima H Minus reverse transcriptase (ThermoFisher, #EP0753), a template switch oligo and an oligodT primer to generate full length cDNA. cDNA was amplified using the Kapa Hotstart 2x ReadyMix (Roche Diagnostics, #7958935001). 1–3 ng cDNA were then tagmentated using 1.3 μg Tn5 and amplified using Kapa HiFi plus dNTPs (Roche Diagnostics, #07958846001) and the following PCR settings: 72 °C 5 min, 98 °C 30 s, 10 cycles of 98 °C for 10 s, 63 °C for 30 s, 72 °C for 1 min, hold at 4 °C. Libraries were quantified using the KAPA library quantification kit (Roche Diagnostis, #079602), and sequenced at PE150 on a NovaSeq 6000 at Novogene.

### 2.6 CUT&Tag library preparation

CUT&Tag was performed as described earlier starting from nuclei [28,29]. First, nuclei were isolated from the sorted macrophages. In short, the macrophages were centrifuged for 5 min at 4 °C, 500 rpm, supernatant was removed, and the cells were lysed on ice in 1 mL of nucleus extraction buffer (1 × prelysis buffer from the EpiGentek EpiQuick Total Histone Extraction Kit, OP-0006-100). To stop the lysis reaction, 1 mL of PBS+1%BSA was added, and nuclei were collected through centrifugation for 5 min at 4 °C, 500 rpm. The supernatant was removed, the nuclei were resuspended in PBS+1% BSA, and a sample was visually inspected for viability, purity, and abundance of nuclei under the microscope. Next, CUT&Tag libraries were created according to the published CUT&Tag protocol for nuclei [29]. All buffers were supplemented with 5 mM sodium-butyrate (Sigma, 303410) and 1X complete protease inhibitor (Merck, 1187358000). Protein lo-bind tubes (Eppendorf, EP0030108116) were used to reduce sample loss. Antibodies against H3K18la (PTM-Bio, PTM-1406), H3K4me3 (Abcam, ab8580), H3K27me3 (Cell Signaling Technology, C36B11) and H3K27ac (Abcam, ab4729) were used in this study. Libraries were indexed using Nextera Indexes, and 150-bp paired-end sequencing was performed on Illumina Novaseq instruments.

### 2.7 Data processing

All genomic data were processed using pipelines built in Nextflow v21.04.3, adapted from the Babraham Institute GitHub repository ([19]) for reproducible data analysis.

#### 2.7.1 RNA-seq

Quality control of the raw sequencing reads was performed using FastQC v0.11.9. Raw reads were trimmed off low-quality bases and adapter sequences using TrimGalore v0.6.6 (https://github.com/FelixKrueger/TrimGalore). Filtered reads were aligned against the reference mouse genome assembly CHRm39 using HISAT2 v2.2.1. Raw gene counts were quantified using the *featureCounts* program of subread v2.0.1.

#### 2.7.2 CUT&Tag

Quality control of the raw sequencing reads was performed using FastQC v0.11.9. Raw reads were trimmed off low-quality bases and adapter sequences using TrimGalore v0.6.6 (https://github.com/FelixKrueger/TrimGalore). Filtered reads were aligned against the reference mouse genome assembly mm10 in case of mouse samples and human genome assembly GRCh38 in case of human samples using Bowtie2 v2.4.4 with options: *-end-to-end --very-sensitive --no-mixed --no-discordant --phred33 -I 10 -X 700*. Aligned bam files were sorted based on chromosomal coordinates using the *sort* function of samtools v1.13. Sorted bam files were summarised into bedgraph files using the *genomecov* function of bedtools v2.30 [30]. Peaks were called from CUT&Tag libraries on individual bedgraph files using SEACRv1.3 in stringent mode with a standard peak calling threshold of 0.01. SEACR is specifically developed for CUT&RUN and is likewise the recommended pipeline for chromatin profiling data with very low background like CUT&Tag. Peaks overlapping with mouse and human blacklist regions were filtered out. Visual QC of bam files and called peaks were performed using IGV.

### 2.8 Data analysis

#### 2.8.1 RNA-seq downstream processing

EdgeR was used for downstream processing of raw gene count matrices. Log-normalised CPM values were calculated after applying TMM normalisation. PCA was performed using the prcomp() function of the R stats package. Differentially expressed genes were defined using FDR <0.05 and abs(log2FC) > 1 as thresholds. Gene clusters were determined empirically through iteration. GO analysis was performed using the R clusterProfiler package v4.4.4.

#### 2.8.2 CUT&Tag downstream processing

Peak sets from both biological replicates were used for downstream analysis. Peaks for each biological replicate were combined to create a master (union) peak list (https://yezhengstat.github.io/CUTTag_tutorial/). This master peak list was used as a reference to generate the fragment count matrix of all samples using the R package chromVAR v1.16. Differential peak analysis was performed as described before [28] using edgeR. Peaks were annotated with the R package ChIPseeker v1.30.3 or overlapped with cCRE regions (see below). Promoter regions were defined as 2000 bp up- and downstream of TSS. Peaks overlapping with promoters were extracted using the annotatePeak function from the R package clusterProfiler v4.0.5 ChIPseeker v1.30.3, selecting only the peaks with promoter annotation for further analysis. The promoter regions were defined using the getPromoters function from the R package ChIPseeker v1.30.3, using the TxDb.Mmusculus.UCSC.mm39.refGene database as input, setting the TssRegion to c(–2000, 2000). For peaks overlapping with PLS/pELS/dELS, they were found using the bedtools function ‘intersect’. The input bed files were the peak bed files together with the cell-agnostic cCRE list bed file available for download for mm10 (https://screen.encodeproject.org/) and converted to GRCm39 using the liftOver function from the UCSC Genome Browser. For dELS-overlapping peaks, their closest, non-overlapping gene was found using the bedtools function ‘closest’. For peaks that overlapped with multiple different cCRE (for instance, 94% of peaks overlapping with pELS also overlapped with the nearest PLS), we created a prioritisation scheme as is done similarly in ChIPseeker. Specifically, the priorities were defined as follows: 1. PLS, 2. dELS, 3. pELS, 4. Other cCREs. For the cistrome transcription-factor binding analysis, the promoter regions of the genes covered by different hPTM combinations were used as input to the online Cistrome database analysis tool using the settings “All peaks in each sample” and “Transcription factor, chromatin regulator”.

#### 2.8.3 Functional enrichment analysis

Gene ontology enrichment analysis was performed using the function *enrichGO* from the R package clusterProfiler v.4.0.5, using the Benjamini-Hochberg *p-*value adjustment method, searching for all ontology categories, using the 3.13.0 versions of org.Mm.eg.db. Comparative GO analysis was performed using the *compareCluster* function from the R package clusterProfiler v.4.0.5 using the same settings.

#### 2.8.4 Data visualization

Principal component plots were generated using the *autoplot* function in the R package ggfortify v.3.48.3 using the output of the *prcomp* function from the R stats v4.3.2 package. All heat maps were generated using the R package pheatmap v1.0.12. Correlation scatter plots were made using the *ggscatter* function from R package ggpubr v0.4. CUT&Tag peak distribution across different genomic features and peak profiles around TSS were visualized using the functions *plotAnnoBar*, and *plotDistToTSS* from R package ChIPseeker v1.30.3. GO analysis results were visualized using the *dotplot* function of the R package enrichplot v.1.12.2. The *boxplot* function from the R package Graphics v4.3.2 was used to plot boxplots.

#### 2.8.5 Statistics

All statistical and other data analyses mentioned above were performed using the statistical programming language R v4.1.0 or above. Group values were compared using two-sided Mann–Whitney *U* tests. Statistical significance was called from (adjusted) *p* < 0.05 or as specified otherwise in the text.

## 3 Results

### 3.1 Macrophages recruited to damaged muscles show a shift in inflammatory status between 2 and 4 days post-injury

**Figure 1:**
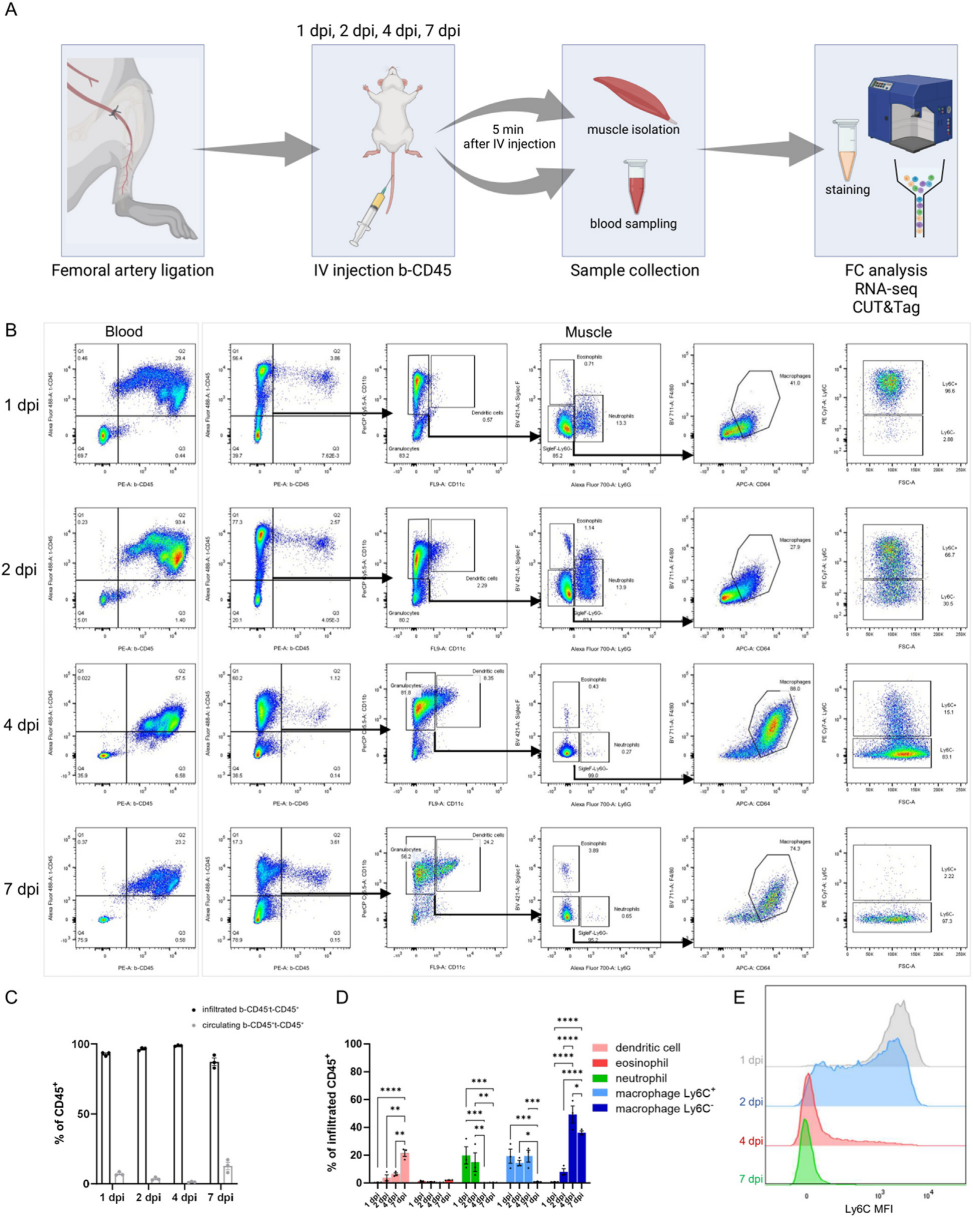
**Profiling of immune cells in muscle after hindlimb ischemia. A)** Schematic illustration of the experimental set-up. Muscle ischemia was induced by the ligation of the femoral artery and macrophages were analysed at 1, 2, 4, 7 dpi. **B)** FACS plots of immune cells in blood and muscle at 1, 2, 4, 7 dpi. **C)** Quantification of circulating/non-infiltrated CD45^+^ cells (b-CD45^+^t-CD45^+^) versus infiltrated CD45^+^ cells (b-CD45^−^t-CD45^+^) at 1, 2, 4, 7 dpi. **D)** Quantification of the different infiltrated b-CD45^−^t-CD45^+^CD11b^+^ populations in the muscle at 1, 2, 4, 7 dpi. **E)** Ly6C expression levels in the macrophage population (F4/80^+^CD64^+^) in muscle at 1, 2, 4, 7 dpi. ANOVA test *p*-values are indicated for each comparison. ∗*p* value < 0.05, ∗∗*p* value < 0.01, ∗∗∗*p* value < 0.001, ∗∗∗∗*p* value < 0.0001.

To study the different immune cell populations implicated during muscle repair after ischemia, we adapted a protocol described in Mysore et al. [24]. Hindlimb ischemia was induced by ligation of the femoral artery [15], and the mice were subsequently sacrificed one, two, four, or seven days post injury (dpi). To differentiate between muscle-infiltrated and circulating immune cells, circulating CD45^+^ cells were labelled *in vivo* through intravenous injection of a FACS-competent antibody 5 min before sacrifice. We refer to the circulating immune cells marked in this way as b(lood)-CD45^+^ cells (Figure 1A,B). After dissection and digestion of the muscles, CD45 was stained a second time with another fluorophore, marking all immune cells present in the digested muscle samples, which we refer to as t(issue)-CD45^+^ and hence comprises both muscle-infiltrated as well as circulating immune cells. To verify the specificity of our antibodies and staining protocol, we also collected blood samples at the time of sacrifice, stained them with the second CD45 fluorophore antibody and confirmed that almost all circulating b-CD45^+^ cells stained positive for CD45 (Figure 1B). More than 90% of all CD45^+^ cells in our samples were muscle-infiltrated (t-CD45^+^b-CD45^-^; 92%, 96%, 98% respectively at 1, 2 and 4 dpi). Only at 7 dpi, the proportion of muscle-infiltrated immune cells decreased to 87% (Fig. 1C).

To further characterise the infiltrated CD45^+^ immune cell population (*i.e.* b-CD45^−^t-CD45^+^), we focused on the different populations of granulocytes as they are the most abundant cell type during the first days of the repair process (>80% at 1, 2 and 4 dpi) (Figure 1B). Eosinophils (CD11b^+^SiglecF^+^) and dendritic cells (CD11b^+^CD11c^+^) were not very abundant, especially at 1, 2, and 4 dpi (the cumulated eosinophil and dendritic cell populations comprise less than 7% of t-CD45^+^ cells, Figure 1D). Neutrophils (CD11b^+^Ly6G^+^) were only appreciably present at 1 dpi (20% of t-CD45^+^ cells) and 2 dpi (15% of t-CD45^+^ cells) and almost absent at 4 and 7 dpi. Macrophages (CD11b^+^F4/80^+^CD64^+^) increased over time post-injury to reach a peak at 4 dpi (around 70% of all t-CD45^+^ cells) before decreasing at 7 dpi to 36% (Figure 1D). All macrophages gradually shifted from a Ly6C^hi^ expressing state at 1 dpi towards a Ly6C^lo^ phenotype with almost no expression by 7 dpi, with the biggest change in Ly6C expression starting at 2 dpi and settling at 4 dpi (Figure 1E). This profile reflects the previously described switch of macrophages from a pro-inflammatory to a restorative phenotype [7,20].

### 3.2 The macrophage transcriptome changes dynamically from one to seven days post ischemia injury

To further characterise macrophage dynamics following hindlimb ischemia, we analysed the transcriptomic profiles of the muscle tissue macrophage population (t-CD45^+^CD11b^+^F4/80^+^CD64^+^) on 1, 2, 4, and 7 dpi. First, we performed a transcriptome-wide comparison with macrophages and monocytes from previous muscle injury studies [7,8]. The gene expression trends of macrophages recruited to ischemia injured muscles reflect those that are observed after CTX-induced muscle injury [7] (Fig. S1A), suggesting conserved macrophage transcriptional dynamics after muscle injury, independently of the cause of injury. Furthermore, a principal component (PC) analysis of an integrated dataset containing all our samples (1–7 dpi), as well as monocytes and macrophages from 1 to 4 dpi in a CTX-induced muscle injury model [8] confirmed this and highlighted the transcriptional differences with monocytes (Fig. S1B). While PC1 largely captured cell type differences, biological differences of the studies, and likely also technical differences, PC2 captured time and state of macrophage polarisation both in our samples as well as the macrophage populations of Patsalos et al. [8]. As also observed in that study, (blood) monocytes clustered away from the macrophages on PC1, highlighting that tissue macrophages have a distinct transcriptional signature from blood monocytes.

**Figure 2:**
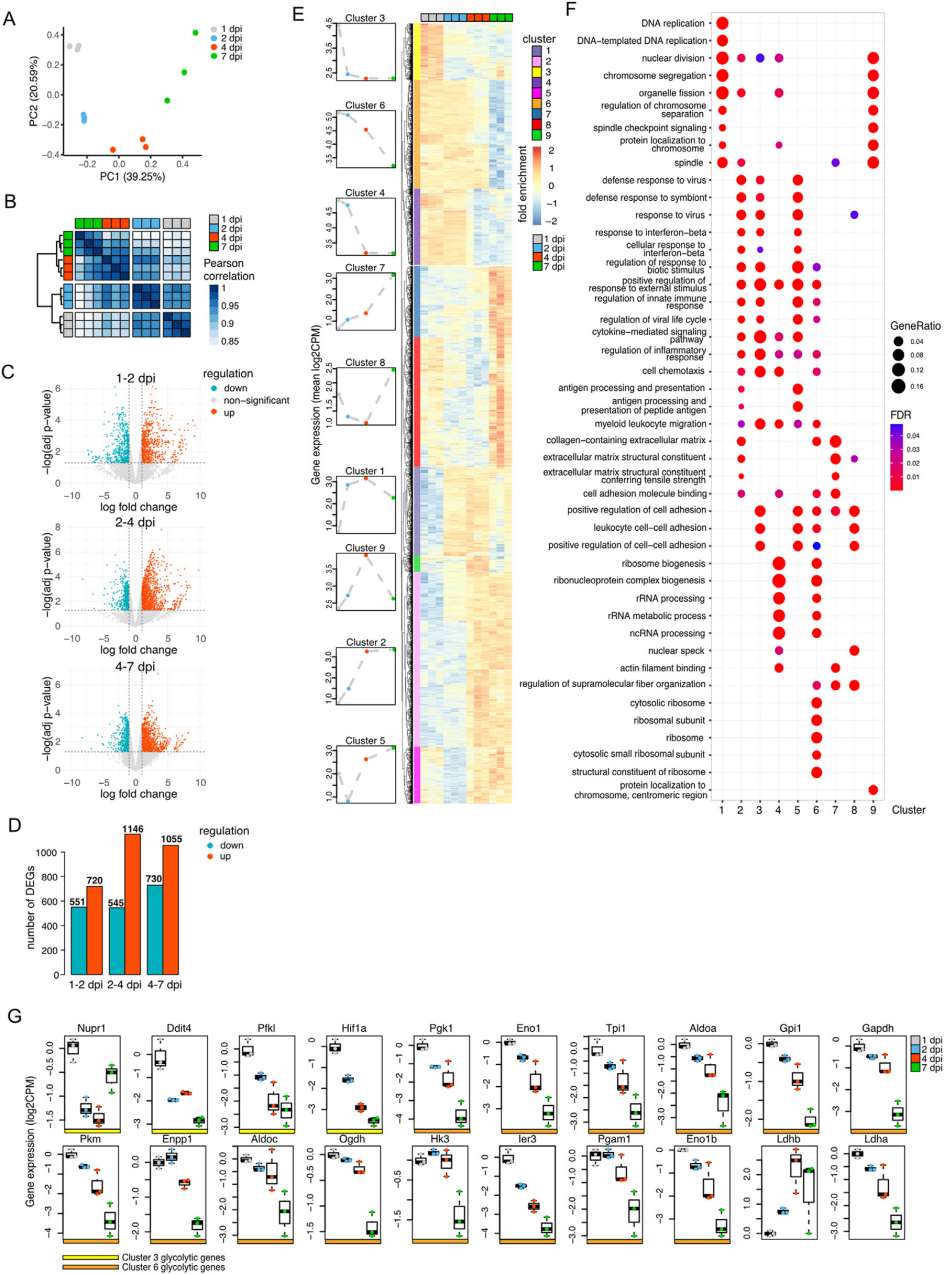
**RNA-seq was used to profile gene expression dynamics in macrophages recruited to the muscle after hindlimb ischemia. A)** PCA of all RNA-seq datasets depicting the first and second principal component. **B)** Pearson correlation and unsupervised hierarchical clustering of normalised gene expression of all samples. **C)** Gene expression changes for all 3 transitions in time are depicted in volcano plots showing the log2FC on the x-axis and -log10 adjusted p-values on the y-axis. Significantly differentially expressed genes (DEGs, defined as abs(log2FC) > 1 and adjusted p-value <0.05) are coloured in red (upregulated) or turquoise (downregulated). **D)** Numbers of DEGs for each transition are depicted as bar plots coloured in red (upregulated) or turquoise (downregulated). **E)** Heatmap of all DEGs from panel D. Unsupervised hierarchical clustering identified 9 clusters with distinct gene expression dynamics in time. The mean expression of each cluster is depicted in the left panels. **F)** comparative GO analysis of all clusters identified in panel E. **G)** Gene expression of genes associated to glycolysis-related GO terms enriched in cluster 3 or 6 and of Ldha and Ldhb, depicted as boxplots containing log2CPM gene expression values.

We then focused on a detailed analysis of our datasets. A PC analysis showed that global gene expression profiles change gradually over the 7-day course post-injury, illustrated by the samples clustering according to and sequential in time along PC1 (Figure 2A). While gene expression at 1 and 2 dpi are still similar, a larger shift can be seen from 2 to 4 dpi and a second shift happens from 4 to 7 dpi (Figure 2A,B). Analysis of differentially expressed genes (DEGs, adj. P < 0.05, log2FC > 1) revealed that many genes are up- or downregulated (Figure 2C,D). We could cluster the DEG dynamics into 9 distinct expression patterns (Figure 2E). Gene Ontology (GO) analysis for all ontologies confirmed that large and coordinated gene expression changes occur during the first week post-injury. Three clusters contain genes with declining expression over time (clusters 3, 4 and 6). Cluster 6 genes decrease gradually over time, cluster 3 genes go down at 2 dpi and cluster 4 genes at 4 dpi. (Pro-)inflammatory processes were the top enriched GO terms for genes that go down at 2 dpi (cluster 3, Suppl. File 1), and also the other two clusters, with gradually downregulated genes (cluster 6) or genes downregulated specifically at 4 dpi (cluster 4) were enriched for pro-inflammatory GO terms, such as ‘myeloid leukocyte migration’, ‘regulation of inflammatory response’ and ‘cytokine-mediated signalling pathway’ (Figure 2F). This is consistent with a downregulation of the proinflammatory Ly6C status over time post-injury (Fig. S1B and [8]) as well as with our own FACS data which shows a shift from Ly6C^hi^ to Ly6C^lo^ that starts at 2 dpi and is complete at 4 dpi (Figure 1). Besides inflammatory GO terms, genes in cluster 4 (downregulated by 4 dpi) were most strongly enriched for RNA and protein synthesis biology (Figure 2F, Suppl. File 1). Genes that gradually go down (cluster 6) were most enriched for cellular bio-energetic terms including several glycolysis terms as well as DNA/RNA/protein-synthesis (Suppl. File 1). Notably, glycolysis-related GO terms such as ‘glycolytic process through glucose-6-phosphate’, ‘regulation of glycolytic process’ and ‘canonical glycolysis’ were only enriched in clusters 3 (downregulated by 2 dpi) and 6 (continuously reducing) (gene expression of the associated genes in Figure 2G), and were absent in any other cluster. Notably, *Ldha* expression goes down gradually in time, while *Ldhb* expression goes up gradually from 1 to 4 dpi, remaining at a high level at 7 dpi (Figure 2G). Two clusters represent genes that increase over time. Cluster 7, which goes up gradually with a strong increase at 7 dpi, is enriched for muscle tissue development and extracellular organisation terms (Figure 2F, Suppl. File 1). Cluster 2, which reaches its peak at 4 dpi is enriched for autophagy, (innate) immune response to IFNβ/virus/symbiont terms (Figure 2F, Suppl. File 1). Four clusters showed dynamic gene expression in time. Cluster 8 first goes down until 4 dpi, after which it strongly increases at 7 dpi and is enriched for muscle tissue development and activation and cell–cell adhesion terms. Cluster 1 goes up until 4 dpi after which it decreases at 7 dpi and is enriched for DNA replication and cell division terms (Figure 2F, Suppl. File 1). Cluster 9 resembles cluster 1, except that its upregulation mostly happens between 2 and 4 dpi instead of between 1 and 2 dpi, and is enriched for cell division (specifically the metaphase and the anaphase) terms (Figure 2F, Suppl. File 1). Lastly, cluster 5 goes down from 1 to 2 dpi after which it increases gradually until 7 dpi and is enriched for antigen processing and presentation and other immune response terms (Figure 2F, Suppl. File 1).

The shift from glycolysis to OXPHOS during macrophage polarisation, which we found represented in gene expression patterns (Figure 1G, Suppl. File 1), has been extensively described before [18]. Importantly, not only lactate generated intracellularly through glycolysis, but also lactate imported from the microenvironment are instrumental during the macrophage phenotypic shift [15,31] and we also observed that lactate in the muscle microenvironment increased significantly after HLI at 1 dpi (from 0.12 nM/μg of tissue at basal condition to 0.25 nM/μg of tissue at 1 dpi) and remained elevated at 3 dpi and 5 dpi (respectively 0.22 nM/μg of tissue and 0.23 nM/μg of tissue) (Fig. S2). Since extracellular lactate levels and glycolysis are known to affect histone lactylation [23], we next explored macrophage histone lactylation dynamics during muscle regeneration as a possible mechanism driving macrophage polarisation.

### 3.3 H3K18la marks active promoters and active enhancers in macrophages

**Figure 3:**
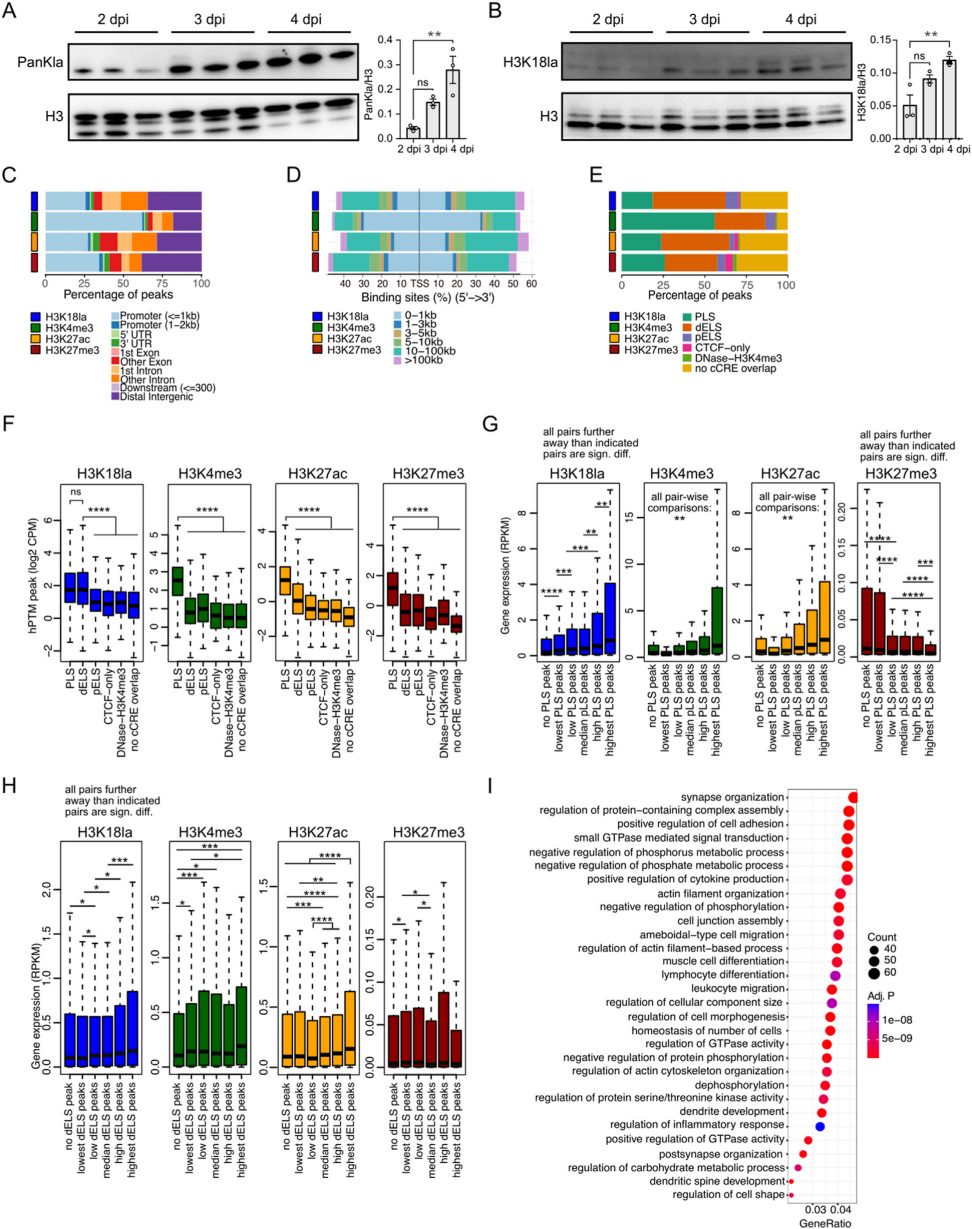
**CUT&Tag was used to profile hPTM genomic distribution dynamics in macrophages recruited to the muscle after hindlimb ischemia. A)** Representative image and quantification of Western blot for PanKla on isolated muscle macrophages after hindlimb ischemia at 2, 3, and 4 dpi. **B)** Representative image and quantification of Western blot on H3K18la on isolated muscle macrophages after hindlimb ischemia at 2, 3, and 4 dpi. **C)** ChIPseeker annotation of hPTMs to genetic elements. **D)** Distance of hPTMs to TSS. **E)** Annotation of hPTMs to cCRE. **F)** hPTM peak levels (log2CPM) depicted as boxplots according to their cCRE-overlap. **G)** Gene expression levels (RPKM) depicted as boxplots according to gene PLS-overlapping peak levels **H)** Gene expression levels (RPKM) depicted as boxplots according to genes closest to dELS-overlapping peak levels **I)** GO analysis of the top 2000 genes closest to dELS covered by the highest H3K18la peaks. All analyses were performed on the master peaks merged for all samples per hPTM with averaged peak counts/values. ANOVA test (A, B) and Wilcoxon test (F, G, H) *p*-values are indicated for each comparison. ∗*p* value < 0.05, ∗∗*p* value < 0.01, ∗∗∗*p* value < 0.001, ∗∗∗∗*p* value < 0.0001.

To evaluate the *in vivo* dynamics of histone lactylation during macrophage polarisation following ischemia-induced muscle injury, we first assessed global histone lactylation levels by western blot. We focused on the period where the macrophages’ inflammatory phenotype switch predominantly takes place and where lactate was shown to be crucial [7,15,20], *i.e.* between 2 and 4 dpi. We found that histones are lactylated both using a pan-lactylation antibody and a H3K18la specific antibody. Moreover, histone lactylation levels increase from 2 to 4 dpi (Figure 3A,B). This result was unexpected, since previously, histone lactylation levels were reported to be lower in M2 (restorative, our 4 dpi) macrophages compared to M1 (inflammatory, our 1–2 dpi) macrophages [23].

To evaluate the potential functionality of histone lactylation towards specific transcriptional changes in the recruited macrophages, we generated maps of H3K18la genomic distributions by CUT&Tag (Fig. S3A). To be able to better interpret these, we also created genomic maps of several key histone PTMs (hPTM), *i.e.* H3K4me3, H3K27ac and H3K27me3, which are well characterised for their relevance in transcriptional control. They respectively mark active promoters, active promoters and active enhancers, or repressed genomic regions.

We called peaks for all hPTMs using the SEACR software which is optimised for CUT&Run and CUT&Tag datasets [32]. hPTM ‘‘peaks’’ represent genomic areas that are enriched for the respective hPTMs. H3K18la peaks span about 1000–2000 bp, which is similar to H3K4me3 (1500–2500 bp) and H3K27ac peaks (1000–3500 bp). H3K27me3 peaks, on the other hand, are considerably larger, spanning between 2000 and 6000 bp (Fig. S3B). We found that H3K18la peaks are less enriched in promoters than H3K4me3 (Figure 3C, Fig. S3C) and more in other regions, at considerable distances (10–100 kb) from the Transcription Start Site (TSS) (Figure 3D, Fig. S3D). Overall, we observed that H3K18la genomic distributions resembled the H3K27ac distribution more than the H3K4me3 distribution. Given that H3K27ac is considered an active enhancer mark (in addition to an active promoter mark), we wanted to investigate how well H3K18la peaks overlap with candidate cis-regulatory elements (cCREs, based on The ENCODE Project Consortium [33]). cCREs represent regions in the genome that have specific regulatory behaviour, such as that of promoters or enhancers. cCREs overlap with DNase hypersensitivity sites, meaning they represent sites of open chromatin. They are defined based on the presence and/or absence of specific hPTMs (H3K4me3, H3K27ac), binding of CTCF and distance to TSS. We regrouped these elements into promoter-like sequences (PLS), proximal enhancer-like sequences (pELS, close to TSS), distal enhancer-like sequences (dELS, far from TSS), CTCF-only regions and H3K4me3/DNase sensitive regions. Although more fine-grained classification of such regulator elements exists (specifying whether they are co-localised with CTCF-binding sites), we did not observe significant differences between such subgroups (CTCF-bound or CTCF-not-bound). Furthermore, due to the physical vicinity of pELS regions to PLS, and their relatively small size (+/−350 bp) compared to the average peak widths, most (>94%) pELS-overlapping H3K18la peaks also overlap nearby PLS, which were annotated to the latter (see methods). We found that only 19% of H3K18la peaks overlapped with PLS, while for H3K4me3 (active promoter) this was 56%, further suggesting that – as compared to the archetype active promoter mark H3K4me3 – H3K18la may be more important in other regions than in promoters. 44% of H3K18la peaks overlap with dELS (Figure 3E, Fig. S3E), which is higher than any other assessed hPTM (41% for H3K27ac peaks). This confirms that H3K18la, besides being an active promoter mark, also marks enhancers [28]. To evaluate its importance to promoter/enhancer activity, we assessed peak strengths (peak max values, peak total values and peak read counts) at different genomic elements and cCRE (Figs. S3F–G, Figure 3F) and found that H3K18la peaks overlapping with dELS were at least as strong as peaks overlapping with PLS (the differences in peak strength variables were not significant) and stronger than those at any other genomic element (p < 2e-16). This was not true for the active H3K4me3 or H3K27ac marks, which showed significantly higher peaks at promoter and/or PLS sites compared to peaks at any other element (p < 2e-16, Figure 3F). Further supporting the importance of H3K18la at enhancers over promoters is the closer resemblance of H3K18la genomic distribution to published enhancer–mark profiles as compared to promoter–mark profiles (Fig. S3H).

Regardless of these findings and although previously demonstrated to also mark active enhancers, H3K18la is primarily described as an active promoter mark [23,28]. Indeed, we found that genes whose PLS is marked by an H3K18la peak were higher expressed than those without a PLS-overlapping H3K18la peak (Fig. S4A). The same is true for the other active marks, H3K4me3 and H3K27ac, but not for the repressive H3K27me3 marks (Fig. S4A). Furthermore, we found that the higher a gene's PLS H3K18la-levels were, the higher their gene expression was, which was also the case for the other active marks, while the opposite was true (high PLS marking, low gene expression) for the repressive H3K27me3 mark (Figure 3G).

Contrary to PLS peaks, which are unambiguously linked to their downstream gene, the gene enhancer (dELS) relationship is less clear and could span large distances. Due to the lack of experimental evidence, we assumed simplified putative dELS-gene interactions. We used an *in silico* approach where we linked dELS to their closest gene, as done also by others [28,34,35]. Similar to PLS-marks, genes closest to H3K18la-occupied dELS were higher expressed than genes closest to dELS without H3K18la occupancy, which we also observed for the other assessed active marks (Fig. S4B). Moreover, gene expression of linked genes increased when dELS H3K18la peaks increased (excluding those that also overlap with PLS) (Figure 3H). Such stepwise increase in gene expression according to dELS H3K18la peak levels was seen to some extent for H3K27ac, but not for H3K4me3. GO analysis of the 2000 genes linked to the top dELS H3K18la peaks confirmed that H3K18la marks enhancers that lie close to genes important to the specific functions of muscle-infiltrating macrophages (Figure 3I, Table S1 for complete GO results, Table S2 for the 2000 genes), including the stimulation of muscle cell proliferation and differentiation, regulation of striated muscle tissue development (Table S3 contains all genes related to terms containing ‘muscle’), lymphocyte differentiation and leukocyte migration.

Since some peaks may overlap with PLS and dELS at the same time and since some genes may have peaks at both their PLS and a close dELS, we also distinguished these from genes that have only a PLS-located peak or only a peak at a nearby dELS. As expected, genes with (a) peak(s) that overlap(s) both PLS and dELS were significantly higher expressed than genes with only a PLS-located peak or only a dELS-peak (for all active marks) (Fig. S4C). Since PLS-peaks can be unambiguously annotated to the corresponding gene while our dELS- closest-gene links are only a simplified approximation, it is expected that PLS-peaks correspond better to gene expression than dELS-peaks (Figure 3G vs Figure 3H, Fig. S4C). In conclusion, we here confirm that H3K18la marks active promoters as well as tissue-specific enhancers. These results are in line with our earlier report on macrophages and other cell types [28], where we have investigated in-depth tissue-specific steady-state H3K18la profiles and its comparison, overlap and coordination to and with other hPTM profiles (including H3K4me3, H3K27ac and H3K27me3) along the genome.

### 3.4 H3K18la dynamics correlate to and predict gene expression dynamics

To better understand the possible function of histone lactylation in macrophages during muscle repair, we decided to investigate whether and which H3K18la changes occur after muscle injury and how they relate to H3K4me3, H3K27ac and H3K27me3 changes. Considering that the biggest shifts in macrophage abundance, inflammatory phenotype (Figure 1 and [7,20]), and gene expression (Fig. 2) occur between 2 and 4 dpi and considering the previously suggested role of histone lactylation for macrophage polarisation [23], we hypothesised that there would be a (functionally) important shift in histone lactylation patterns between 2 and 4 dpi. While hypothesised to be instrumental for the switch from a pro-inflammatory to a restorative phenotype [23], no previous study has investigated how histone lactylation profiles change upon adaptation of a restorative phenotype.

We did not observe large qualitative changes with regards to the overall genomic distribution of H3K18la peaks (Figs. S3C–E), or to that of H3K4me3 and H3K27ac peaks. Repressive H3K27me3 peaks were slightly less present in promoters at 4 dpi compared to 2 dpi. Only 10.5% of H3K18la peaks called at 2 dpi were not detected in any 4 dpi H3K18la peak set and only 4.2% peaks called at 4 dpi were not detected at 2 dpi. Furthermore, only 7.7% of the 3281 promoters that had a H3K18la peak at 2 dpi were not marked by a H3K18la-peak at 4 dpi and only 2.9% of the 3157 promoters that were marked by a H3K18la-peak at 4 dpi were not marked by a H3K18la-peak at 2 dpi. None of both groups were enriched for a specific GO term.

**Figure 4:**
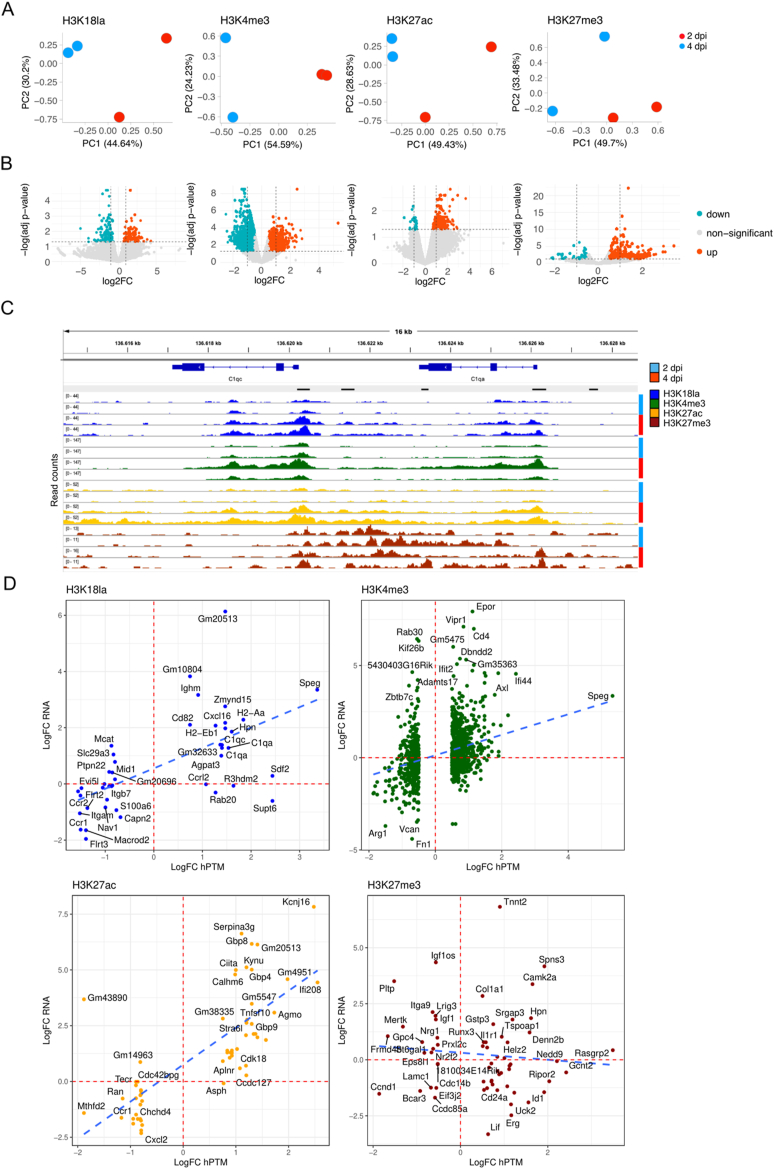
**H3K18la dynamics correlate to/predict gene expression dynamics.A)** PCA of quantified peaks of each hPTM. **B)** Volcano plots depicting hPTM changes between 2 and 4 dpi. On the x-axis, log2FC are depicted, on the y-axis, -log10 adjusted P-values are depicted. Significantly hyper- and hyporegulated peaks are defined as having a log2FC > 0.5 and log2FC < (−0.5), respectively and an adjusted P-value <0.05. Hyperlactylated peaks are depicted in red and hypolactylated peaks are depicted in turquoise. **C)** Snapshot of IGV view depicting all profiled hPTM genomic distributions at a region containing hyperlactylated peaks at the PLS of *C1qa* and *C1qc*. Each track is group normalised together with the other tracks from the same hPTM. **D)** Scatterplots showing log2FC gene expression changes on the y-axis and log2FC of hPTM peaks covering the gene-associated PLS on the x-axis. Only pairs with a significantly changed peak are depicted. Blue dotted lines indicate the linear regression for the gene expression versus hPTM changes. Red dotted lines indicate the baseline x- and y-axes.

Next, to investigate whether quantitative changes occurred, we computed a master peak file combining all 4 peak sets (n = 2 for 2 dpi and n = 2 for 4 dpi) per hPTM which was quantified in all 4 samples of the respective hPTM. PC analysis confirmed that the biological replicates from 2 dpi were more similar to each other than to the replicates from 4 dpi, and this for all 4 hPTMs, indicating that robust quantitative changes did occur for all 4 hPTMs (Figure 4A). Using stringent cut-off criteria (log2FC > 0.5 and FDR <0.05) to define differentially marked regions, we found 141 hyperlactylated and 159 hypolactylated regions from 2 to 4 dpi (Figure 4B, Fig. S5A, example in Figure 4C). The genomic distribution of these hyper- and hypolactylated peaks over various cCREs was comparable to that of the overall H3K18la peak distribution (Fig. S5B). Genes with significantly increased H3K18la PLS marking (and gene expression, see further) (n = 20, 10 of which have their PLS covered by the same peak that also covers the PLS of another gene and 10 of which do not share a PLS-peak with another gene, Table S4) were enriched almost exclusively in GO terms (n = 30) related to lymphocyte mediated immunity (Table S5, Fig. S5C).

Sixteen out of the 20 genes with significantly increased H3K18la PLS-overlapping-peaks showed concordant upregulation in their expression from 2 to 4 dpi (Figure 4D, Fig. S6). As expected, such concordance between a stronger PLS-localised peak signal and its associated gene's overexpression was also observed for the other active hPTMs, H3K27ac and H3K4me3 while the opposite was seen for the repressive H3K27me3 mark (Figure 4D). Four genes (Supt6, Ccrl2, Rab20 and R3hdm2) did not follow this pattern, but these four genes showed increased expression from 4 to 7 dpi (Fig. S6), indicating that the increase in PLS-localised H3K18la may be instructive for future gene expression induction. Furthermore, the PLS of two of these genes (Supt6 and Rab20) were covered by increased H3K18la-peaks that also covered the PLS of an adjacent gene that did have correlating elevated gene expression (Sdf2 and E230013L22Rik, respectively) (Figs. S5D and E). For a third gene, R3hdm2, the increased H3K18la peak overlapped with one of its 6 PLS as well as with a nearby dELS, which is where the actual H3K18la-increase appeared to happen (Fig. S5F). Ccrl2, the fourth of these genes with increased H3K18la PLS-marking but no change in gene expression (log2FC = −0.014, P = 0.95), also had increased H3K4me3 (log2FC = 0.55, P = 5.4E-05) and H3K27ac (log2FC = 0.65, P = 0.0014) PLS-marking.

Such mechanisms where H3K18la promoter increases anticipate later gene expression increments were also described in the original paper describing histone lactylation [23]. We therefore correlated all significant hPTM PLS-peak changes from 2 to 4 dpi to gene expression changes from 1 to 2 dpi (Fig. S7A) and from 4 to 7 dpi (Fig. S7B) and found that H3K18la PLS changes indeed correlate better with future gene expression changes than to past gene expression changes. When evaluating the gene expression profiles of genes that have hyperlactylated promoters from 2 to 4 dpi, 18 (out of 20) genes show increased gene expression from 4 to 7 dpi (Fig. S6). On the other hand, 12 (out of 20) genes with hyperlactylated promoters from 2 to 4 dpi showed a downregulation in expression from 1 to 2 dpi. This indicates that H3K18la primes for gene expression changes later in time rather than following gene expression changes earlier in time. This observation was not (or to a much lesser extent) true for other active hPTMs (Fig. S7). Furthermore, not all genes that were differentially expressed earlier in time (from 1 to 2 dpi), also have consequential differential H3K18la PLS marking from 2 to 4 dpi (Fig. S8A), indicating that altered H3K18la levels are not a consequence of altered gene expression. In fact, for any set of differentially expressed genes (earlier in time, at the same time or later in time Figs. S8B–C), only a subset also had differential PLS H3K18la levels, suggesting that H3K18la PLS changes are selective. Altogether, these results indicate that H3K18la may be instructive for regulating the expression of specific genes, rather than being a consequence of altered gene expression.

In fact, among all upregulated DEGs from 2 to 4 dpi (n = 1146, Figure 2D), only a small subset had concurrent increased H3K18la PLS levels (338, of which 15 significant), which is far below that for H3K4me3 (637, of which 144 significant) or H3K27ac (576, of which 24 significant). Among all downregulated DEGs (n = 545, Figure 2D), also only a small subset had decreased H3K18la PLS levels (144, of which 4 significant), *i.e.* lower than H3K4me3 (308, of which 76 significant) or H3K27ac (400, of which 11 significant). To investigate coordinated action of the various hPTMs in relation to gene expression, we compared the DEGs to hPTM PLS changes. We overlapped all up- or downregulated DEGs with genes with significantly hyper- or hypo-hPTM-marked PLS's, respectively, and found only few overlapping pairs (Fig. S8D). Next we correlated PLS hPTM-changes pair-wise, specifically for the DEGs (Fig. S8E). These correlations show that H3K18la and H3K4me3 and H3K27ac are actually pair-wise strongly positively correlated (pearson R between 0.41 and 0.66, all p < 2.2e-16) and the majority of upregulated genes has increased combinations of active PLS-hPTM-peaks (represented by the upper-right quadrant; 50.23% for the H3K18la-H3K4me3 pair, 46.16% for the H3K4me3-H3K27ac pair and 46.31% for the H3K18la-H3K27ac pair). Correspondingly, the majority of downregulated genes has decreased combinations of active PLS-hPTM-peaks (represented by the lower-left quadrant; 60.78% for the H3K18la-H3K4me3 pair, 74.29% for the H3K4me3-H3K27ac pair and 59.49% for the H3K18la-H3K27ac pair).

Lastly, to investigate if hPTM PLS changes are related to genes with specific biological functions or differ much as to which pathways are affected, we performed a comparative GO enrichment analysis of the genes with significantly differentially enriched PLS peaks, comparing the different hPTMs.

It is important to note that almost all genes with significantly increased H3K18la PLS-peaks also have an increased H3K4me3 PLS peak (although often non-significant), but that the opposite is not true: not all significantly increased H3K4me3 PLS peaks have matching increased H3K18la PLS-peaks (Fig. S9A). Similarly, almost all genes with significantly increased H3K27ac PLS-peaks also have an increased H3K4me3 PLS peak, but not all significantly increased H3K4me3 PLS peaks have matching increased H3K27ac PLS-peaks (Fig. S9A). Interestingly, almost all genes with significantly increased H3K18la PLS-peaks also have an increased H3K27ac PLS peak and the opposite is also true: almost all significantly increased H3K27ac PLS peaks have matching increased H3K18la PLS-peaks. In addition, comparative GO analysis (Fig. S9B) showed that it is the subset of genes with increased H3K18la as well as H3K27ac and (although not always significant) H3K4me3 that is enriched for macrophage-specific functions during 2–4 dpi. Genes that have increased H3K4me3 PLS levels but not matching increased H3K18la and/or H3K27ac levels on the other hand were not enriched for macrophage-specific functions. This analysis therefore supports the idea that H3K18la is a more selective hPTM when it comes to regulating macrophage-specific biology as compared to H3K4me3.

## 4 Discussion

Previous *in vitro* work has shown that lactate drives histone lactylation and gene expression in macrophages, promoting the expression of a specific set of `restorative' genes within inflammatory macrophages [23]. The role of histone lactylation during *in vivo* macrophage functional reprogramming, has remained unexplored and its distribution in restorative macrophages has not been described. The dynamic reprogramming of macrophages from a pro-inflammatory Ly6C^hi^ towards a restorative Ly6C^lo^ phenotype during skeletal muscle regeneration is well characterised [1,7,15,20,36]. Here we have investigated the accompanying transcriptional and epigenetic changes of macrophages that are recruited to muscles upon ischemic injury from 1 to 7 dpi. Comparing the transcriptional changes in macrophages upon ischemia-induced muscle injury with previous work in the field that analysed transcriptional changes after CTX-induced injury showed that both injuries result in similar transcriptional changes, suggesting a conserved macrophage response upon muscle injury, independent of its cause [7,8]. We however cannot exclude that the specific setting of hypoxia in this arterial ligation-induced injury model, might differently affect macrophage metabolism and/or genetic drivers of macrophage repolarization.

When investigating the potential function of H3K18la for macrophage biology, we chose to profile it in both inflammatory and restorative macrophages and to compare its genomic distribution to several reference hPTMs that are well characterised. Importantly, by creating the data for both H3K18la and the reference hPTMs in-house, in the same study, using the same samples, we made sure that they are comparable to each other, originating from cells that were subjected to the exact same conditions. Epigenetic profiles are specific to the cell type and cell state, and are known to change when cells are exposed to different environments and/or stimuli. Therefore, comparing H3K18la distributions to that of reference hPTMs obtained from the same cell populations allowed us to better estimate H3K18la genomic enrichment and potential function.

In our analysis, we did not robustly detect novel peaks from 2 to 4 dpi or vice versa. Relaxing or tightening peak calling thresholds could result in the discovery of more or less unique peaks, but will inevitably also result in more or less false or true positive results.

A crucial step in the investigation of the potential function of epigenetic modifications, is their link to (and effect on) transcriptional dynamics. Our study is the first to create H3K18la profiles for inflammatory as well as for restorative macrophages and to also include corresponding RNA-seq data. Linking epigenetic changes to transcriptional changes is relatively straightforward for epigenetic changes occurring at gene promoters (PLS). The relation between a gene's promoter epigenetic state and its transcriptional output is very well described for a plethora of epigenetic modifications, including the hPTMs used here as references, and our findings are in line with literature. We observed that increased H3K18la PLS marking matches future gene expression changes better than past or concurrent gene expression changes. In fact, similar observations were made in *in vitro* polarised macrophages where promoter H3K18la changes of M0 versus M1 macrophages appeared most specifically at pro-restorative genes (n = 6), for instance of *Arg1* which is typically expressed at the end of M1-polarisation (24h exposure to LPS-IFNy), and not at early-induction inflammatory genes (expressed after 4h–8h exposure to lPS-IFNy) [23]. However, none of the 6 example restorative genes from Zhang et al.‘s study was recovered in the top hits from our study and also the time-line of macrophage polarisation was considerably different. These discrepancies between both studies are most likely the result of the different models, notably using an *in vivo* versus *in vitro* system to study macrophage polarisation, altered lactate production kinetics or levels, altered lactate sources (intracellular only versus intra- and extracellular), and/or the focus on different phenotypic transitions: M0/M1 H3K18la changes versus 2 dpi (inflammatory)/4 dpi (restorative) H3K18la changes.

As shown here, as well as in various other cell and tissue types [28,35], H3K18la appears to be an important active enhancer mark that is dynamically changing during cell state transitions. H3K18la was the only hPTM investigated (including the known active enhancer mark H3K27ac) for which enhancer signals were as strong as promoter signals. Linking enhancers to their target genes is not as simple as linking promoters to their target genes. Empirically determining genome-wide enhancer-target gene-links requires complex Hi-C experiments which comprehensively characterise genome-wide chromatin interactions. The outcomes of those experiments are cell type and time point specific, due to constantly changing enhancer–gene interactions. Therefore, we opted for an *in silico* approach where we linked enhancers to their closest gene, without overlapping with its promoter region, which has limitations, but is often used as an approximation to estimate the effect of enhancer activity on gene expression [28,34,35]. Using this strategy, we found that H3K18la marks enhancers linked to genes important for macrophage-mediated muscle regeneration (being enriched for terms such as lymphocyte differentiation and muscle cell differentiation). Furthermore, we also found that gene expression increases stepwise with higher H3K18la enhancer peaks, which was not the case for the other hPTMs. Together, these findings indicate that dynamic H3K18la marking at both enhancers and promoters instructs future gene expression changes of macrophages to convert from a pro-inflammatory to a pro-restorative phenotype.

The contribution of glycolysis and/or glycolysis-induced lactate secretion in macrophage biology has received significant attention. High glycolysis and lactate production is a key feature of inflammatory macrophages which directly links metabolism to inflammatory cytokine production and macrophage migration [19]. Lactate also affects the polarisation of macrophages towards a restorative phenotype and contributes to orchestrating macrophage effector functions. But whether glycolysis-derived lactate or lactate that is taken up from the environment drives alterations in histone lactylation, is not clear. In cancer for instance, lactate uptake from the tumour microenvironment promotes macrophage polarisation through stabilising the HIF-1a transcription factor [31]. In the ischemic skeletal muscle, lactate levels rapidly rise [15,37,38] (Fig. S2). This rise is fast and occurs before the arrival of monocytes and their differentiation to macrophages, but lactate levels remain high for several days after ischemia. The cellular origin of this surge in lactate levels is unclear. While the hypoxic muscle fibers likely are the main source of lactate, we have surprisingly measured that also angiogenic endothelial cells, which are highly glycolytic and upregulate glycolysis further during the formation of new blood vessels following ischemia, contribute to the rise of muscle lactate levels [15]. On similar note, the high number of glycolytic neutrophils and macrophages in the hypoxic muscle few days after induction of ischemia (where meanwhile many fibers are dying or are dead - likely reducing the production of lactate from fibers), potentially affects lactate levels inside the muscle. The exact contribution of different cell types is however difficult to unravel given existing lactate shuttles and extensive previous work showing that inhibition of lactate production in cells might affect their function and/or metabolism via alternative mechanisms [31,[39], [40], [41]]. Focusing on macrophages, we showed that under *ex vivo* conditions, they can take up lactate and that this contributes to their repolarization towards reparative macrophages. Also blocking MCT1 (*Slc16a1*) -dependent lactate uptake prevented the repolarization of macrophages *in vivo*, but the underlying mechanism was not resolved [15]. On the other hand, in the hypoxic muscle, newly recruited pro-inflammatory macrophages need high glycolysis and generate lactate themselves. For instance, efferocytosis-induced lactate production induces the proliferation of restorative macrophages through its receptor GPR132 [42]. At the same time, lactate also enhances the expression of efferocytosis receptors MerTK and LRP1 to boost continuous efferocytosis via an intracellular Ca^2+^-dependent mechanism [43]. Manipulation of lactate levels via increasing glycolysis-dependent lactate production or by increasing extracellular lactate availability could affect macrophage gene regulatory networks and function either through metabolic regulation, or through metabolic control of epigenetic marks and at the same time affect histone lactylation. We observed detectable levels of histone lactylation at 2 days after ischemia induction in macrophages (that likely arrived less than 24 h before harvesting), and lactylation levels increased by day 4. Between day 2 and day 4, we already observed decreasing expression of glycolytic genes, including *Ldha* versus an increase in *Ldhb* (see Figure 2G). Based on those observations and knowing that lactate levels are high as soon as monocytes enter the ischemic tissue, we can only speculate that main changes in lactylation could be driven by extracellular lactate levels. However, given potential delays between glycolysis and lactylation *in vivo*, mechanisms of cellular crosstalk etc, and the lack of clear evidence supporting this hypothesis, future studies using novel tools to affect lactylation independent of other (metabolic) effects of lactate will be required to univocally show the critical contribution of lactylation for macrophage function, and its potential for therapeutic targeting.

## 5 Conclusion

In this work, we show that macrophages recruited to the muscle after ischemic injury modify their histone lactylome between 2 and 4 days post injury. Absolute histone lactylation levels increased and also, although subtly, the genomic enrichment of H3K18la changed from 2 to 4 days post injury. This suggests that changes in lactylation are functional and not the mere consequence of altered lactate availability. Interestingly, we found that alterations in H3K18la genomic enrichment from day 2 to day 4 post injury are predictive for gene expression changes later in time, from day 4 to day 7, rather than being a reflection of past gene expression changes from day 1 to day 2. Future studies using novel tools to affect lactylation independent of other (metabolic) effects of lactate will be required to univocally show the critical contribution of lactylation for macrophage function.

## Funding

Open access funding provided by 10.13039/501100003006Swiss Federal Institute of Technology Zurich. This work was funded by the 10.13039/100000001Swiss National Science Foundation (310030_208041), by ETH Zurich core funding, a post-doctoral fellowship to EG by the Future Food Initiative, a program run by the World Food System Center of ETH Zurich, the Integrative Food and Nutrition Center of EPFL, and their industry partners. KDB is endowed by the Wilhelm Schulthess Foundation.

## CRediT authorship contribution statement

**T. Desgeorges:** Writing – review & editing, Writing – original draft, Visualization, Validation, Methodology, Investigation, Funding acquisition, Formal analysis, Data curation, Conceptualization. **E. Galle:** Writing – review & editing, Writing – original draft, Visualization, Validation, Software, Methodology, Investigation, Funding acquisition, Formal analysis, Data curation, Conceptualization. **J. Zhang:** Investigation, Methodology. **F. von Meyenn:** Writing – review & editing, Writing – original draft, Validation, Supervision, Funding acquisition, Conceptualization. **K. De Bock:** Writing – review & editing, Writing – original draft, Validation, Supervision, Resources, Project administration, Investigation, Funding acquisition, Conceptualization.

## Declaration of competing interest

The authors declare that they have no known competing financial interests or personal relationships that could have appeared to influence the work reported in this paper.

## Figures and Tables

**Figure:**
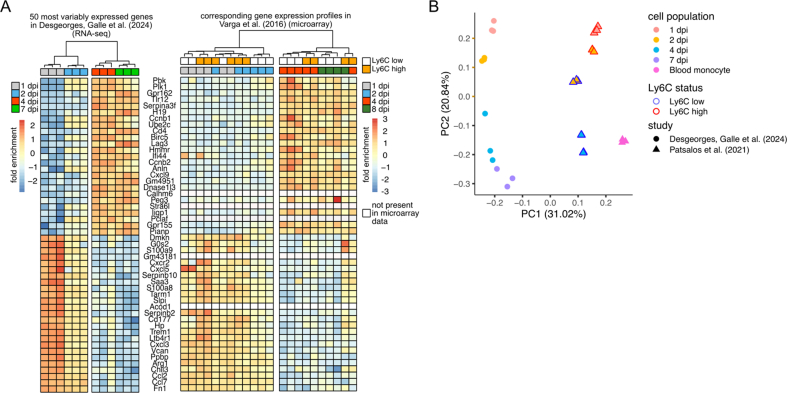
Supplemental Figure 1. Quality check of the RNA-seq data as compared to previously published datasets obtained from similar biological sources. **A**) gene expression of the 50 genes with most variable expression in our data, depicted as heatmaps for our data (left) and published microarray data obtained from a CTX-injury model (right). Enrichment values are normalised row-wise. Missing genes (n = 4) in the microarray data are omitted (depicted by white boxes). Samples were clustered automatically according to similarity. Genes were ordered based on average expression on 1 dpi in our data. Clustering was performed according to the Ward.D2 method in R. **B)** PCA of the RNA-seq datasets from our study integrated with that of Patsalos et al. (2022) [8]. The raw reads were combined and processed as 1 big dataset to obtain logCPM counts using the R package edgeR. PCA was calculated on the normalised dataset using the R package stats' function *prcomp*.

**Figure:**
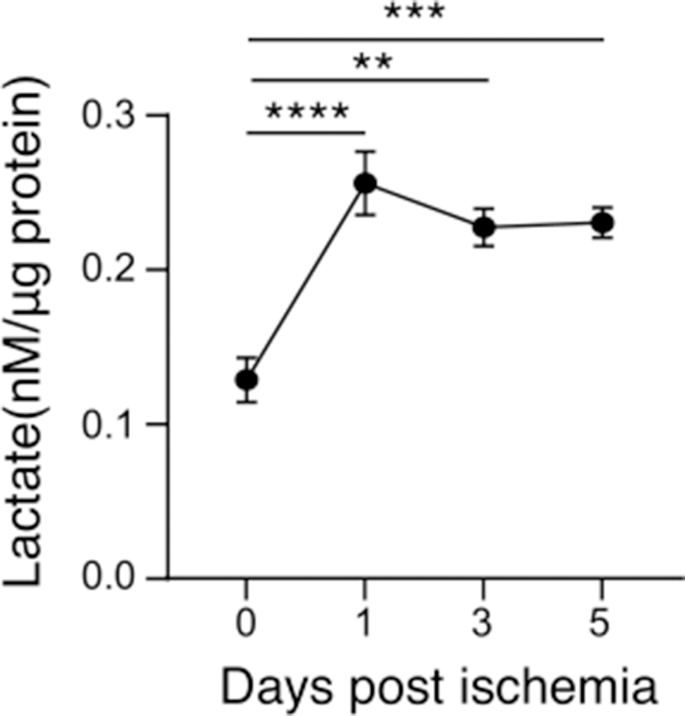
Supplemental Figure 2. Lactate measurement in muscle before (0 dpi), 1, 3, and 5 days post injury (dpi). ANOVA test *p*-values are indicated for each comparison. ∗∗*p* value < 0.01, ∗∗∗*p* value < 0.001, ∗∗∗∗*p* value < 0.0001.

**Figure:**
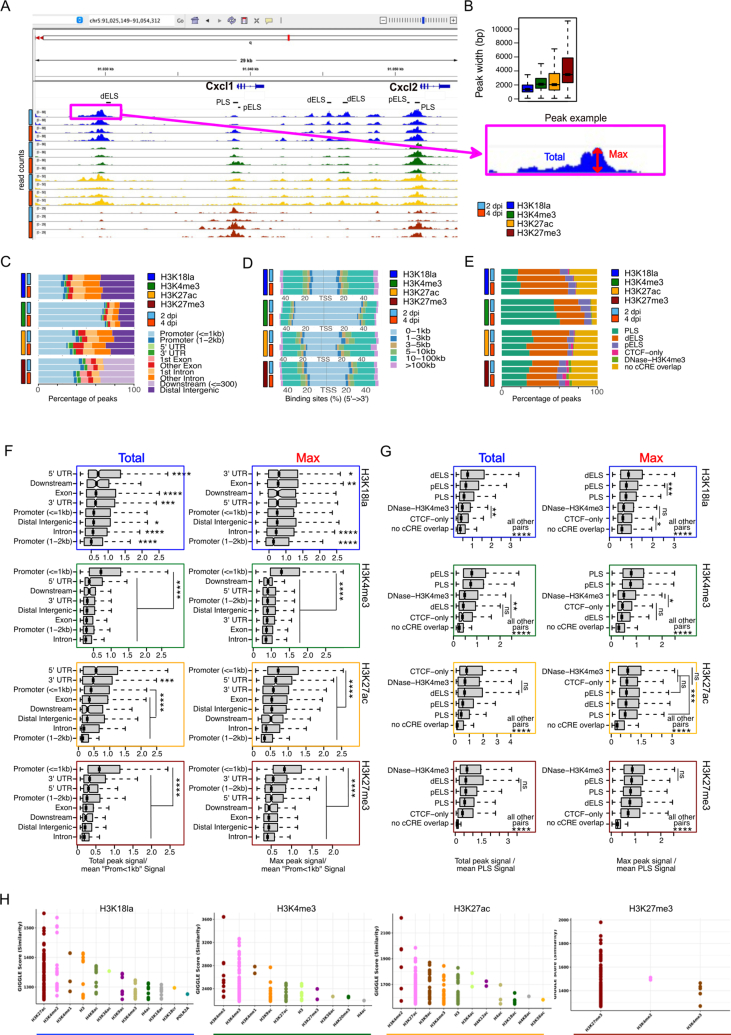
Supplemental Figure 3. Profiling of hPTM genomic distribution dynamics by CUT&Tag in macrophages recruited to the muscle after hindlimb ischemia. **A**) Snapshot of IGV view depicting all profiled hPTM genomic distributions. Each track is group normalised together with the other tracks from the same hPTM. An example of max and total peak values is depicted in a zoomed-in window. **B)** Boxplots showing peak widths of all peaks, separated per hPTM. **C)** ChIPseeker annotation to genetic elements of each sample. **D)** Distance of each sample's distribution to TSS. **E)** Annotation to cCRE of each sample's genomic distributions. **F)** hPTM peak values (total and max) depicted as boxplots according to their genomic element annotation. Boxplots are ordered according to decreasing median value. Significance is calculated between each element and the Promoter (<1 kb) element. **G)** hPTM peak values (total and max) depicted as boxplots according to their cCRE-overlap. Boxplots are ordered according to decreasing median value. **H)** Similarity of our hPTM distributions to published hPTM distributions assessed using the Cistrome database [44]. For panels F, G and H, the master peaks were used, with values averaged over all input samples. Wilcoxon test *p*-values are indicated for each pair-wise comparison. ∗*p* value < 0.05, ∗∗*p* value < 0.01, ∗∗∗*p* value < 0.001, ∗∗∗∗*p* value < 0.0001.

**Figure:**
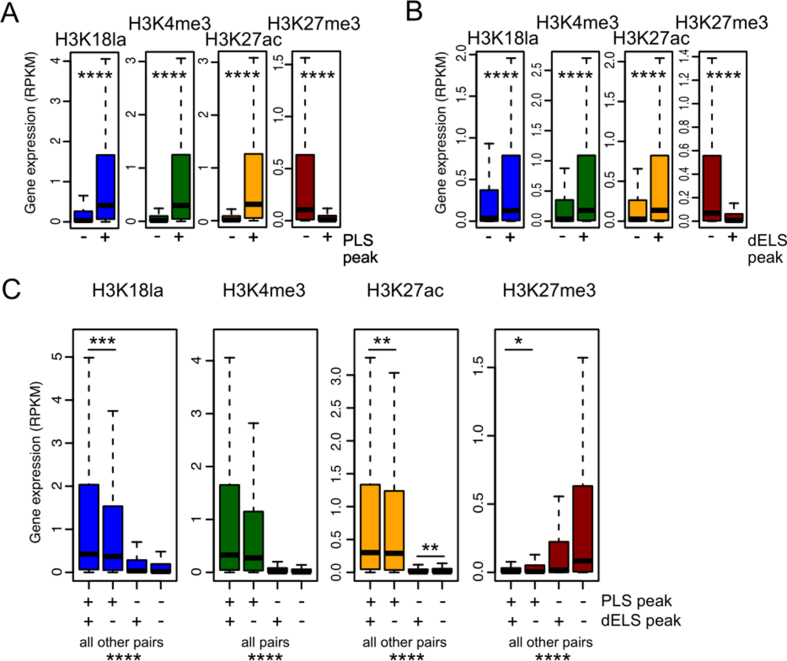
Supplemental Figure 4. Gene expression stratified for PLS and dELS peak occupancy. **A**) Gene expression levels (RPKM) depicted as boxplots according to gene PLS hPTM occupancy. **B)** Gene expression levels (RPKM) depicted as boxplots according to the closest dELS peak occupancy. **C)** Gene expression levels (RPKM) depicted as boxplots according to gene PLS and/or dELS hPTM occupancy. Wilcoxon test *p*-values are indicated for each pair-wise comparison. ∗*p* value < 0.05, ∗∗*p* value < 0.01, ∗∗∗*p* value < 0.001, ∗∗∗∗*p* value < 0.0001.

**Figure:**
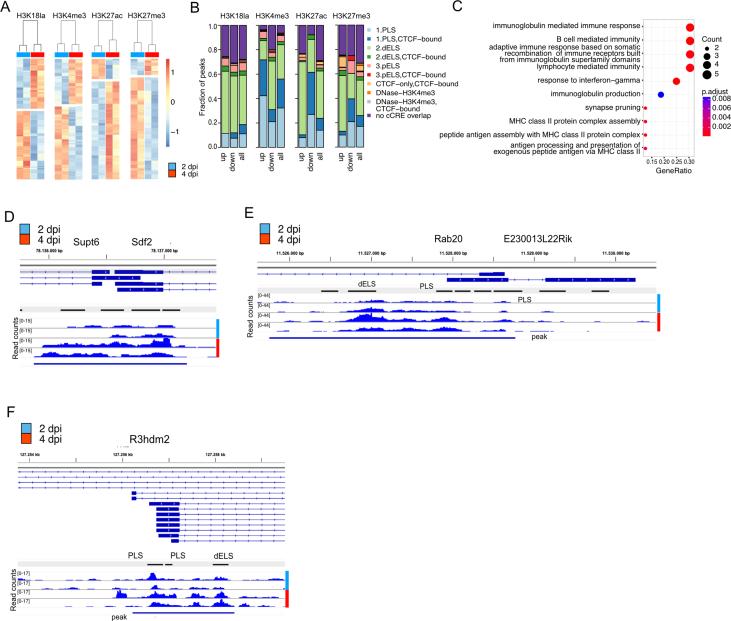
Supplemental Figure 5. H3K18la dynamics from 2 to 4-day post injury. **A**) Heatmaps depicting significantly changed peaks from 2 to 4 dpi (abs(log2FC) > 0.5 & FDR<0.05). Unsupervised clustering separated hyper- and hypolactylated peaks as well as samples from 2 or 4 dpi. **B)** Fractional overlap of significantly changed hPTM peaks (abs(log2FC) > 0.5 & FDR<0.05) as well as all peaks with cCRE. If a peak overlapped with 2 different cCRE, it was annotated to the cCRE with the highest rank (defined according to the order indicated in the panel). **C)** GO enrichment of significantly hyperlactylated peaks overlapping with PLS. **D-E)** Examples of hyperlactylated peaks overlapping the PLS's of 2 different genes, only one of which has concordant upregulated gene expression. **F)** Example of an hyperlactylated peak overlapping both a PLS of a gene without concordant upregulated gene expression and a dELS.

**Figure:**
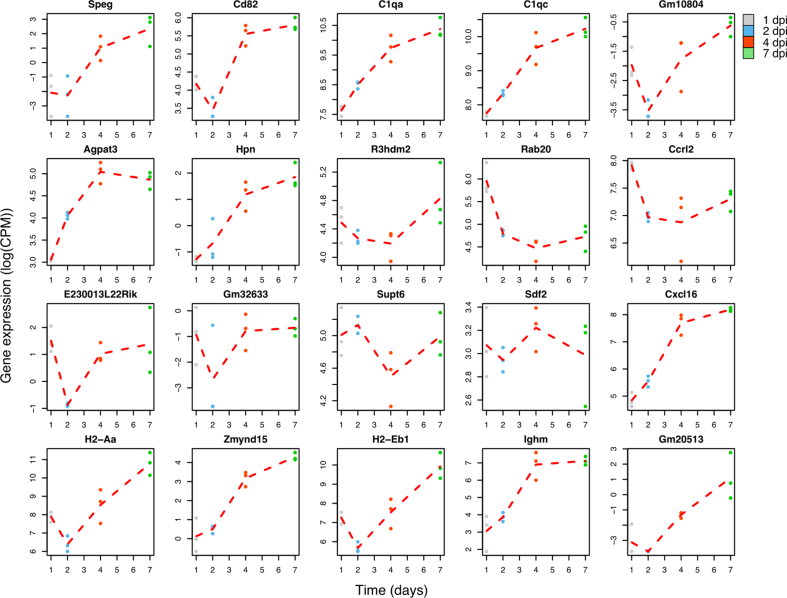
Supplemental Figure 6. Gene expression dynamics over time of genes with hyperlactylated promoters. Dot plots depicting gene expression (y-axis, log(CPM)) of all genes with significantly increased PLS-localised H3K18la peaks in time (x-axis, days). A red dotted line indicates the average gene expression profile.

**Figure:**
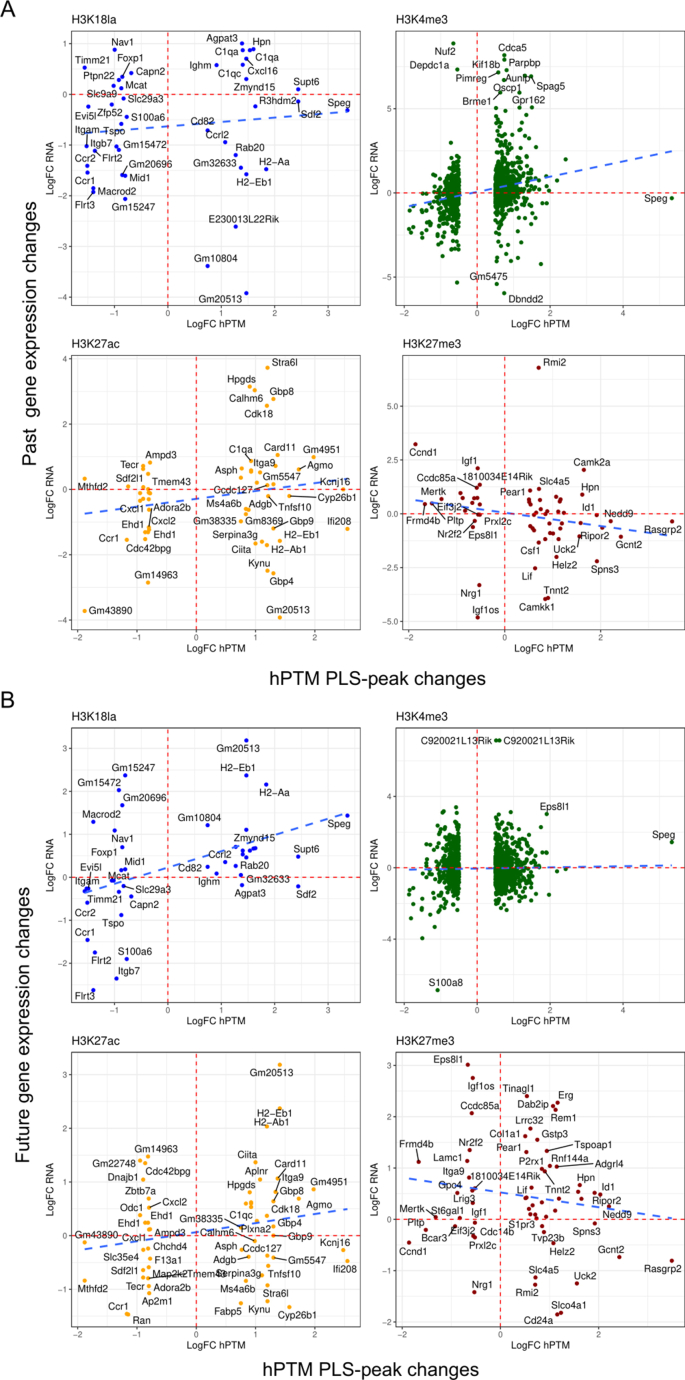
Supplemental Figure 7. H3K18la dynamics correlate to or predict gene expression dynamics. **A**) Scatterplots showing log2FC gene expression changes from 1 to 2 dpi on the y-axis and log2FC of hPTM peaks covering the gene-associated PLS from 2 to 4 dpi on the x-axis. Only pairs with a significantly changed peak are depicted. Blue dotted lines indicate the linear regression for the gene expression versus hPTM changes. Red dotted lines indicate the baseline x- and y-axes. **B)** Scatterplots showing log2FC gene expression changes from 4 to 7 dpi on the y-axis and log2FC of hPTM peaks covering the gene-associated PLS from 2 to 4 dpi on the x-axis. Only pairs with a significantly changed peak are depicted. Blue dotted lines indicate the linear regression for the gene expression versus hPTM changes. Red dotted lines indicate the baseline x- and y-axes.

**Figure:**
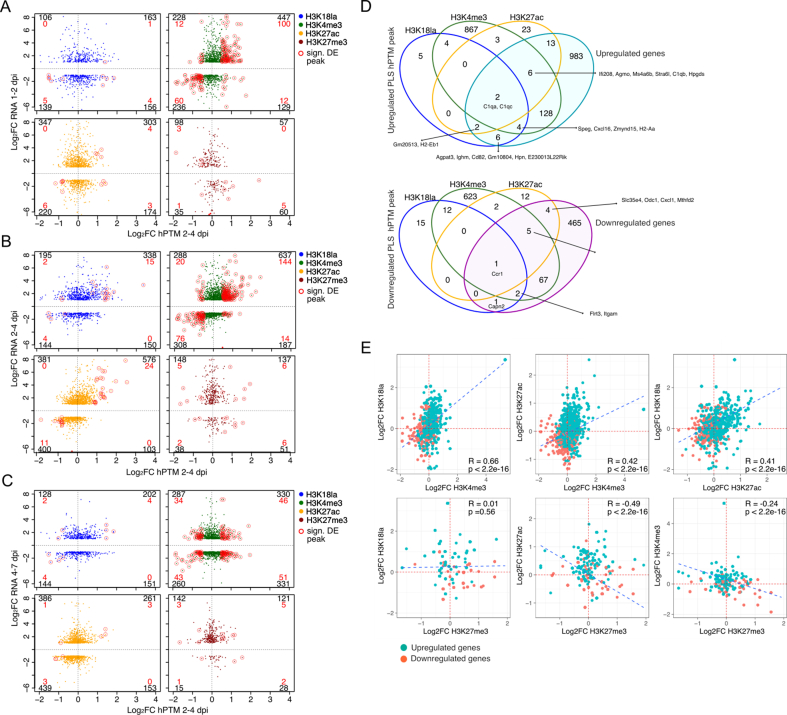
Supplemental Figure 8. The interaction between PLS-localised H3K18la-, H3K27ac-, H3K4me3-and H3K27me3-changes and gene expression changes. **A**) Scatterplots showing (past) log2FC gene expression changes from 1 to 2 dpi on the y-axis and log2FC of hPTM peaks covering the gene-associated PLS from 2 to 4 dpi on the x-axis. Only pairs with significantly changed gene expression are depicted. Dotted lines indicate the baseline x- and y-axes. Significantly DE peaks are indicated in red circles. Numbers in each quadrant indicate the total number of dots (DEGs) in that quadrant. Red numbers indicate, per quadrant, the number of DEGs with a significant DE PLS peak. **B)** Scatterplots showing (contemporary) log2FC gene expression changes from 2 to 4 dpi on the y-axis and log2FC of hPTM peaks covering the gene-associated PLS from 2 to 4 dpi on the x-axis. Only pairs with significantly changed gene expression are depicted. Dotted lines indicate the baseline x- and y-axes. Significantly DE peaks are indicated in red circles. Numbers in each quadrant indicate the total number of dots (DEGs) in that quadrant. Red numbers indicate, per quadrant, the number of DEGs with a significant DE PLS peak. **C)** Scatterplots showing (future) log2FC gene expression changes from 4 to 7 dpi on the y-axis and log2FC of hPTM peaks covering the gene-associated PLS from 2 to 4 dpi on the x-axis. Only pairs with significantly changed gene expression are depicted. Dotted lines indicate the baseline x- and y-axes. Significantly DE peaks are indicated in red circles. Numbers in each quadrant indicate the total number of dots (DEGs) in that quadrant. Red numbers indicate, per quadrant, the number of DEGs with a significant DE PLS peak. **D)** Vennplots showing the overlap between up- or down-regulated genes and genes with hyper- or hypo- (active) hPTM-marked PLS, in upper and lower panels, respectively. **E)** Pairwise correlation of 2 hPTM promoter-overlapping peak log2FC's. Each dot is 1 DEG. The x-axis depicts the peak level log2FC of a peak overlapping with the gene promoter of hPTM 1 and the y-axis depicts the peak level log2FC of a peak overlapping with the gene promoter of hPTM 2. The dots are coloured according to their gene expression regulation (significantly up- or downregulated at FDR <0.05 and abs(logFC) > 1). Only DEGs (gene expression change FDR <0.05 and abs(logFC) > 1)) are depicted. Blue dotted lines indicate the linear regression between the hPTM changes. Red dotted lines indicate the baseline x- and y-axes.

**Figure:**
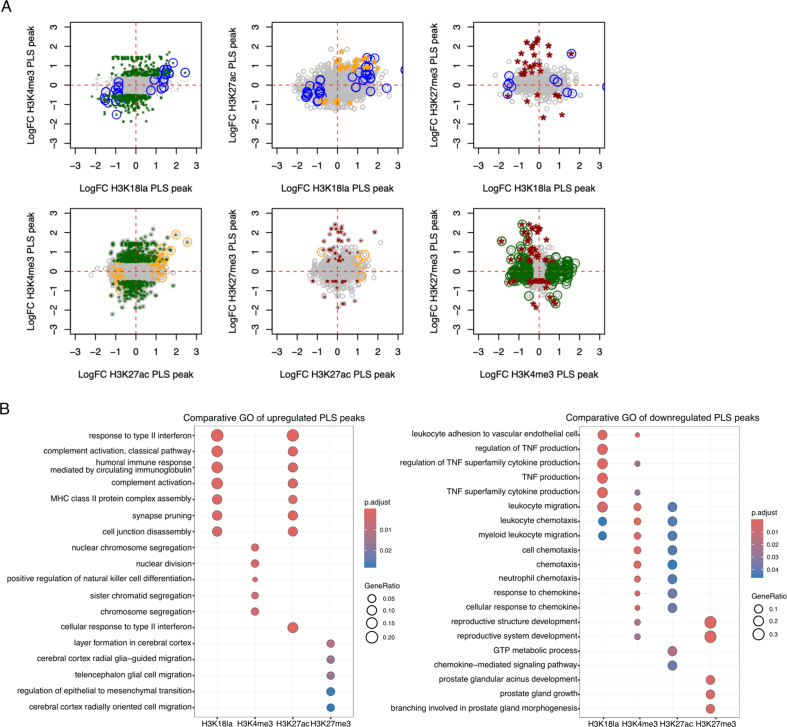
Supplemental Figure 9. The correlation between PLS-localised H3K18la-, H3K27ac-, H3K4me3-and H3K27me3 changes and Gene Onthology associated pathway. **A**) Pair-wise correlation of hPTM PLS-level changes. x- and y-axes are showing the log2FC of hPTM peaks overlapping PLS. Each dot represents the log2FC of 2 hPTMs at the PLS of 1 gene. Significantly DE peaks are highlighted in blue (H3K18la), green (H3K4me3), orange (H3K27ac) or red (H3K27me3). Red dotted lines indicate the baseline x- and y-axes. **B)** Comparative GO enrichment of genes with increased hPTM peaks at their PLS, calculated using the compareCluster function of the R package clusterProfilerv4.10.0. The top 5 enriched terms per hPTM are shown.

## Data Availability

Data will be made available on request.

## Appendix A Supplementary data

Supplementary Table 1. GO analysis of the 2000 genes linked to the highest dELS H3K18la peaks. List of Gene Ontology terms that are enriched among genes linked to the highest dELS H3K18la peaks. The table is the direct result from the enrichGO function from the clusterProfiler package (see methods).

Supplementary Table 2. genes linked to the highest dELS H3K18la peaks. Ensembl identifiers, gene names and entrez identifiers of the top 2000 genes that are linked to the highest dELS H3K18la peaks

Supplementary Table 3. Subset of genes related to muscle terms in Table S1.

Supplementary Table 4. Genes with hyperlactylated PLS. List of genes that have hyperlactylated peaks (from 2 to 4 dpi) overlapping with their PLS.

Supplementary Table 5. GO analysis of genes with hyperlactylated PLS. List of Gene Ontology terms that are enriched among genes that have hyperlactylated peaks (from 2 to 4 dpi) overlapping with their PLS. The table is the direct result from the enrichGO function from the clusterProfiler package (see methods).
