# Supplementary figures and images for: Histone lactylation in macrophages is predictive for gene expression changes during ischemia induced-muscle regeneration
Source: Mol Metab. 2024 Mar 22;83:101923. doi: 10.1016/j.molmet.2024.101923 (PMC11002880; doi:10.1016/j.molmet.2024.101923)

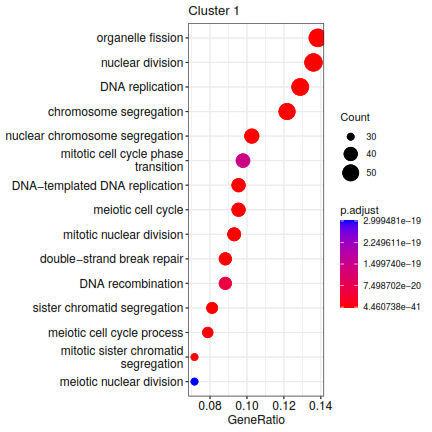

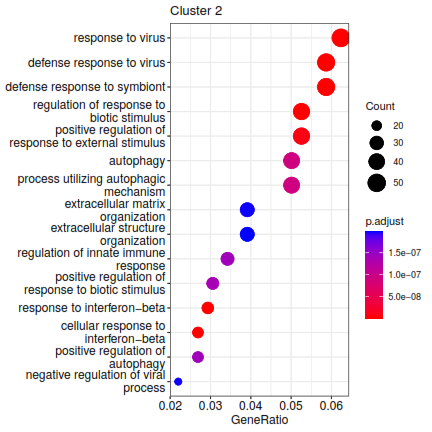

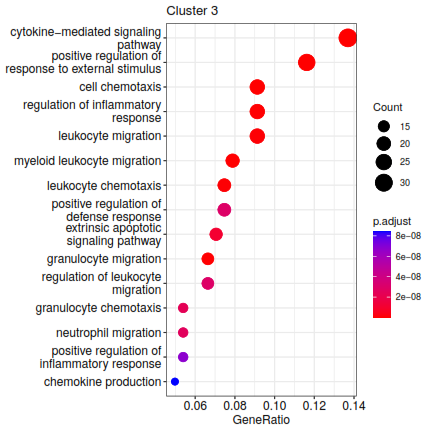

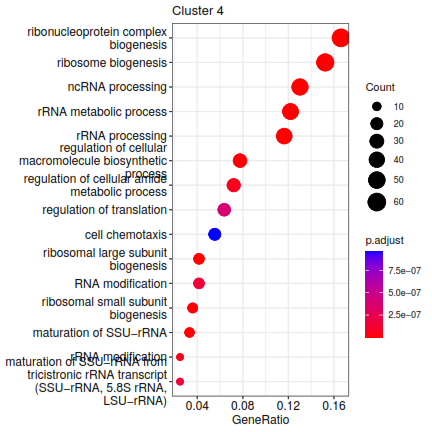

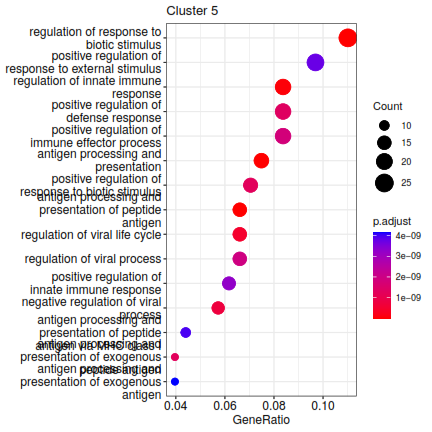

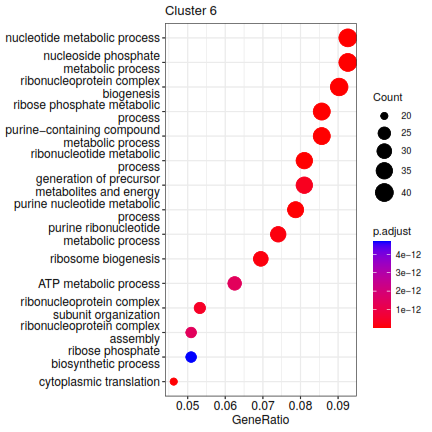

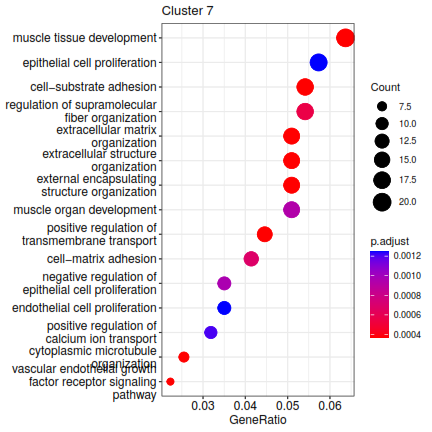

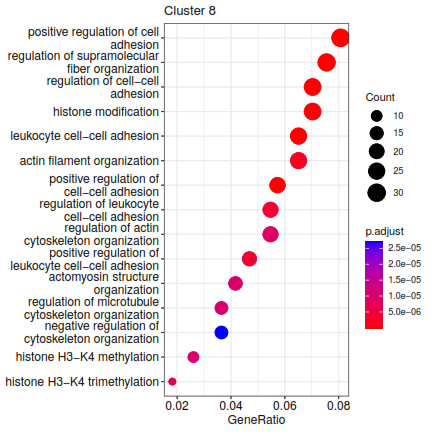

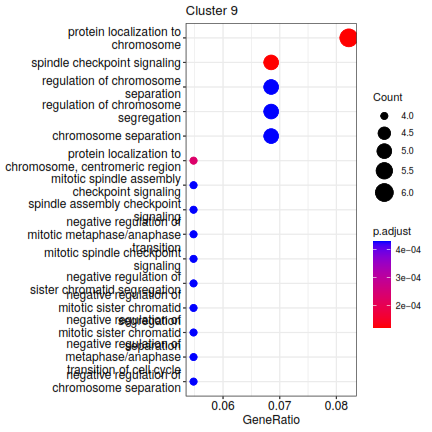

Supplement: Supplemental File 1 [file mmc6.docx]
